# A Case Study on the Development of a Data Privacy Management Solution Based on Patient Information

**DOI:** 10.3390/s20216030

**Published:** 2020-10-23

**Authors:** Arielle Verri Lucca, Luís Augusto Silva, Rodrigo Luchtenberg, Leonardo Garcez, Xuzeng Mao, Raúl García Ovejero, Ivan Miguel Pires, Jorge Luis Victória Barbosa, Valderi Reis Quietinho Leithardt

**Affiliations:** 1 Laboratory of Embedded and Distribution Systems, University of Vale do Itajaí, Rua Uruguai 458, C.P. 360, Itajaí 88302-901, Brazil; arielle@edu.univali.br (A.V.L.); luisaugustos@usal.es (L.A.S.); rodrigol@edu.univali.br (R.L.); leonardo.conceicao@edu.univali.br (L.G.); 2 Expert Systems and Applications Lab, Faculty of Science, University of Salamanca, Plaza de los Caídos s/n, 37008 Salamanca, Spain; xuzengmao@usal.es; 3 Expert Systems and Applications Lab., E.T.S.I.I of Béjar, University of Salamanca, 37008 Salamanca, Spain; raulovej@usal.es; 4 Instituto de Telecomunicações, Universidade da Beira Interior, 6200-001 Covilhã, Portugal; impires@it.ubi.pt; 5 Computer Science Department, Polytechnic Institute of Viseu, 3504-510 Viseu, Portugal; 6 UICISA:E Research Centre, School of Health, Polytechnic Institute of Viseu, 3504-510 Viseu, Portugal; 7 Applied Computing Graduate Program, University of Vale do Rio dos Sinos, Av. Unisinos 950, São Leopoldo, RS 93.022-750, Brazil; jbarbosa@unisinos.br; 8 Departamento de Informática da Universidade da Beira Interior, 6200-001 Covilhã, Portugal; 9 COPELABS, Universidade Lusófona de Humanidades e Tecnologias, 1749-024 Lisboa, Portugal; 10 VALORIZA, Research Center for Endogenous Resources Valorization, Instituto Politécnico de Portalegre, 7300-555 Portalegre, Portugal

**Keywords:** data privacy, taxonomy, IoT, COVID-19

## Abstract

Data on diagnosis of infection in the general population are strategic for different applications in the public and private spheres. Among them, the data related to symptoms and people displacement stand out, mainly considering highly contagious diseases. This data is sensitive and requires data privacy initiatives to enable its large-scale use. The search for population-monitoring strategies aims at social tracking, supporting the surveillance of contagions to respond to the confrontation with Coronavirus 2 (COVID-19). There are several data privacy issues in environments where IoT devices are used for monitoring hospital processes. In this research, we compare works related to the subject of privacy in the health area. To this end, this research proposes a taxonomy to support the requirements necessary to control patient data privacy in a hospital environment. According to the tests and comparisons made between the variables compared, the application obtained results that contribute to the scenarios applied. In this sense, we modeled and implemented an application. By the end, a mobile application was developed to analyze the privacy and security constraints with COVID-19.

## 1. Introduction

Internet of Things (IoT) devices can be applied in various sectors, acting as a facilitating tool [1]. Devices may help monitor health conditions without the presence of healthcare professionals [2]. There are also wireless technologies that monitor older adults and remotely send data such as heart rate and blood pressure to their caregivers [3]. In addition to monitoring, other devices have auxiliary functions, such as automatic insulin injection devices [4]. These are directly linked to sensitive patient data and provide additional control in critical situations by, for example, setting the dose to be injected into the insulin pump. Both privacy settings and control information must have an extreme level of security.

For hospital environments, IoT devices are distributed not only for patient use but also for other functionalities. According to Farahani et al. [5], some of the IoT applications used in hospital settings collect patient data, such as heart rate, blood pressure, or glucose level. As far as the environment is concerned, some sensors detect temperature changes or control the air conditioning; cameras are used to detect intruders and send alerts. In this context, the devices’ scope ranges from patient monitoring to evaluate the environment and the equipment used by health professionals. Thus, the data is recorded from the moment that patients are registered at the reception until they are discharged.

When the patient is registered for admission, basic information is collected and complemented after screening. In a first-aid environment, to ensure all patients’ safety, many hospitals use a screening technique known as the Manchester Protocol [6]. After screening, the information is added to the patient’s record. Next, the person is given a classification according to their condition; this varies from non-urgent cases to emergency intervention cases. Sensitive information is added to the user record, whose preservation and confidentiality level must be treated as critical. There is information that should not be disclosed or related to the patient, as is the case with a patient suspected of having viral and infectious diseases.

The current pandemic of Severe Acute Respiratory Syndrome Coronavirus 2 (COVID-19 SARS-CoV-2) causes the patient to be identified as a possible carrier even during the screening process, based on certain symptoms. According to Rothan and Siddappa [7], those infected usually show symptoms after approximately five days, the most common signs of illness being fever, cough, and fatigue; the patient may also present headaches, phlegm, hemoptysis, diarrhea, shortness of breath, and lymphopenia. These symptoms are identifiable without specific examinations that are directly documented in the patient’s medical record. Liang et al. [8] mention that for most patients diagnosed with COVID-19, 85.7% had fever, 42.9% had cough, 33.3% had expectoration, 57.1% had fatigue and 38.1% had headache and dizziness. For this reason, one can see that fever is a common symptom. Thus, this condition must be checked as soon as the patient is admitted to the hospital. Due to COVID-19’s high rate of contagion, the patient’s referral to medical care and subsequent isolation should be done quickly and strictly in confirmation.

When it is confirmed that the patient has a COVID-19 infection, this information is directly linked to their record, which should remain confidential. Soares and Dall’Agnol [9] comment that privacy is considered an individual right that includes the protection of the intimacy of the subjects, respect for dignity, limitation of access to the body, intimate objects, family and social relationships. In addition, in this same bias, the concern also covers the complete information collected during the patient care process. Even though patients’ data must be confident among all parties in general, due to the current pandemic situation and contagion rate, an extra precaution must be taken to join the statistics without having their information revealed. The application of privacy on patient data must be given to all levels with access to any information, be it registration, device, or image.

The main purpose of this work is to apply privacy constraints in patients with suspected COVID-19. The basis for the application of privacy is the same for patients in general, but using as a basis the fact that it is a pandemic situation, and the discretion in handling data of a suspected patient is crucial. Also, as it is a highly contagious virus, the process from admission to the emergency room to the patient’s referral must be done quickly. In this way, a taxonomy was proposed that covers four topics and five subtopics regarding the entities/environments participating in the hospital admission process.

The scientific contribution of this paper is a system to support the privacy constraints related to COVID-19. It started with the study of the state-of-the-art in hospital environment. Next, we defined a taxonomy, and a mobile application was implemented to test and validate the use of the mobile application to cover the privacy constraints defined in the taxonomy.

The main results of this study are related to the identification of the users. Cryptography methods were implemented control the users according to the diagnosis of COVID-19. As these data are related to health, it must be secure and anonymous. The data collected included reliable data related to temperature parameters for the detection of the symptoms, such as fever.

For a better understanding of the matter and a clearer overview of the relevant details, this work is organized as follows: Section 2 lists the related works; Section 3 describes the taxonomic definition developed for this project and the attributes of the user parameter, environment, privacy, and device; Section 4 demonstrates the modeling of the project, including the use cases, sequence and context diagrams; in Section 5, we present the prototype with the application developed to be validated. Section 6 presents experiments and results. Finally, in Section 7, we conclude and discuss the future work.

## 2. Related Work

Studies on the application of privacy in hospital settings cover different aspects. Various studies were selected to identify privacy targeting, including encryption, profile privacy, device privacy, and taxonomic definitions. The focus among the related papers vary from studies on security over mobile application to systems conceived to protect user privacy.

Barket et al. [10] present a broad study on the context of privacy, developing a taxonomy meant to connect privacy and technology based on the following aspects: purpose, visibility, and granularity. According to the authors, the aim is related to why the information is requested; depending on the cause, more or fewer details about the user are passed on. Visibility refers to who is allowed to access the user data. Granularity designates the data transfer required for the type of access and purpose for that particular request.

The work of Asaddok et al. [11] involves mobile devices in the area of health (Mobile Health (mHealth) and the parameters: usability, security, and privacy. The authors propose a taxonomy that involves the three parameters mentioned, and, for each, it branches into taxonomies. One taxonomy is defined by usability, effectiveness, efficiency, satisfaction, and learning. Next, for security, confidentiality, integrity, and availability is restricted to another taxonomy. Finally, for privacy, identity, access, and disclosure, the last taxonomy is defined.

Coen-Porisini et al. [12] describe a conceptual model for defining privacy policies that cover the user, the user’s profile, the information, and the action that will be taken by a third party to request the information. The authors revealed the link between the three topics mentioned in a Unified Modeling Language (UML) format. The user is divided into personnel—the person to whom the data is referred; processor—the person who will request the data; controller—the person who controls the actions requested by the processor. Data is divided into: identifiable—in situations when it is clear who the data refers to, such as the name; sensitive—it refers to information, processing, and purpose. We can also observe that there is an interaction between the medical user and the controller, along with the processes of access (processing), treatment (purpose), and communication (obligation). The diagram demonstrates how information is delivered to the medical user through requests, based on their access profile.

Silva et al. [13] use a notification management system focused on user privacy in this context. It contributed to the development of an application that can handle different types of notifications. Moreover, the network made it possible for those involved to ensure that the messages sent and received followed the rules defined earlier. If applied to health notifications or to alert cases of COVID-19, this is a strategic tool, addressing messages with defined priorities while also linking privacy in the traffic sent. Therefore, this work contributes to finding a link between IoT requirements and definitions. In [14], the authors implemented a system for monitoring and profiling based on data privacy in IoT. From the results obtained in the tests, they identified different profiles assigned to random situations. In this case, the health system user’s profile priorities would apply and determine which profiles would be authorized to receive data. In this work, it was also possible to address the evolution and reduction of the hierarchy based on factors that identify users’ frequency in the environments tested.

Concerning the relationship between data privacy and its use in situations such as the COVID-19 crisis, Zwitter et al. [15] deals with the basic concept of human rights that relates data privacy with the need to use certain information, such as someone’s location. The authors mention features of applications developed by China, South Korea, and the United States that use tracking techniques to indicate close contact with virus carriers or identify specific individuals or groups’ movements. The study concludes that location data is important in the fight against the spread of the virus, but other relevant information, such as genetic data, should be considered. It is necessary to use this information correctly, as stipulated by the law. It also states that data sensitivity classification is contextual; data protection and privacy are important and must be maintained even in crisis. Information leaks are inevitable, so organizations should always protect themselves; ethics in data manipulation is mandatory for more efficient analysis.

Yesmin et al. [16] deal with the privacy of patients’ data in terms of the interoperability of systems and the employees’ access to information. Also, they tell us that there is no framework for evaluating privacy audit tools in hospitals yet. The application of a framework would help identify any trend in accessing the data and allow the hospital to improve its performance in detecting possible data leaks. According to the authors, the literature reveals that the most significant leakage of information occurs through employees (nurses, doctors, sellers, and others). An evaluation framework was then developed and tested using the black box concept, which uses usability testing information. The following must be monitored through machine learning or artificial intelligence tools: employee access to information, validation of entry and non-standard behavior, and unexplained access to files.

The work of Islam et al. [17] deal with a survey on the application of IoT devices in the health system. The authors deal with the IoT network’s topology for health, which facilitates the transmission and reception of medical data and enables data transmission on demand. They also mention features of wearable devices, which capture and store patient data. These may include blood sugar levels, cardiac monitoring, body temperature, and oxygen saturation. The authors explain that the security requirements applied to healthcare IoT equipment are similar to those of other communication scenarios. Therefore, the following must be considered: confidentiality, integrity, authentication, availability, data update, non-denial, authorization, resilience, fault tolerance, and fault recovery.

Sun et al. [18] designed the HCPP (Healthcare System for Patient Privacy) system to protect privacy and enable patient care in emergency cases. The entities defined for the system are the patient, the doctor, the data server, the family, the personal device, and the authentication server. According to the authors, the system meets the following security criteria: privacy, data preservation by backup, access control, accountability, data integrity, confidentiality, and availability.

Samaila et al. [19] developed a survey in which information was collected regarding work on security and privacy in IoT in general. The study’s scope ranges from security, encryption, communication protocols, authentication to privacy, among others. The authors also collected information on applications, reliability, and other technical issues, combining ten related works. Additionally, the authors claim that the work covers a system model, a threat model, protocols and technologies, and security requirements. The work discusses the IoT architecture considering nine application domains: home automation, energy, developed urban areas, transport, health, manufacturing, supply chain, wearables, and agriculture. Security measures and system and threat models were defined for each application domain, including protocols and communications. The security properties covered were confidentiality, integrity, availability, authenticity, authorization, non-repudiation, accountability, reliability, privacy, and physical security. These also describe mechanisms that can be applied to achieve the desired security requirements: authentication, access control, encryption, secure boot, security updates, backup, physical security of the environment, and device tampering detection.

Plachkinova, Andrés and Chatterjee [20] elaborated a taxonomy focused on privacy over mHealth apps. Downloadable apps through the app store do not have a unified way to provide terms of use or privacy policies for the user. Apps mostly communicate between patients and doctors, access to patient medical records, self-diagnosis based on symptoms, etc. The management of user data after the app is installed may not be precise. The authors elaborated a taxonomy that embraces the following three dimensions: mHealth app (patient care and monitoring; health apps for the layperson; communication, education and research; physician or student reference apps), mHealth security (authentication; authorization; accountability; integrity; availability; ease of use; confidentiality; management; physical security) and mHealth privacy (identity threats; access threats; disclosure threats).

Alsubaei, Abuhussein, and Shiva [21] proposed a taxonomy aiming to enhance security among IoT medical devices, as it has life-threatening risks when a device is not secure. According to the authors, since security and privacy are becoming challenging due to the sensitivity of data in healthcare, it is crucial to enhance these measures. The taxonomy is based on the following topics: IoT layer, intruders, compromise level, attack impact, attack method, CIA compromise, attack origin, attack level, and attack difficulty. For each topic, some subsections embrace items from that topic. Since new attacks are always being created, this taxonomy can be updated, according to the authors. The related works we have selected cover the topics that we cited as critical to privacy. Some applied cryptography in the study as a reference of types of attacks, and others used cryptography to prevent data from being accessed from third parties. Most of them applied user profile privacy to prevent any unauthorized access or mitigate when it happens.

Data encryption is necessary so that in the event of an attack, a third party cannot gain access to information [22]. Cryptography is part, directly, from [17,18]. Islam et al. [17] mentioned cryptography among security threats, where cryptographic keys can be stolen to collect user sensitive data. The work of Sun et al. [18] mentioned encryption as a way to protect health information and applied identity-based cryptography for encryption, authentication, and deriving shared keys for their Healthcare system for Patient Privacy (HCPP) protocols. Also, they made use of searchable symmetric encryption to return encrypted documents to the owner.

The application of private profile was mentioned in all works, except by [21]. The user’s profile privacy serves to protect any information from being used by third parties [23]. A security layer should be applied at the device level to prevent third parties from accessing information or even gaining control of it [24]. The work of Alsubaei, Shiva, and Abuhussein [21] mentions about attacks that influences on Confidentiality, Integrity and Availability (CIA) triad, which is a basic thread on privacy, but does not explore ways to protect user privacy concerning data access based on authorization. Barker et al. [10] are concerned about private profile through who can access the data and which data can be accessed, based on the purpose of this access request.

Asaddok and Ghazali [11] defined data access based on access to patient identity information, personal health information, and personal health records, moreover defined in their taxonomy as identity, access, and disclosure. Coen-Porisini et al. [12] say that data access must be based on access control based on the users and their roles. Thus, data access must be granted based on a consent given by the patient. Silva et al. [13] defined their privacy requirements based on the user permissions, environment, and hierarchy. Leithardt et al. [14] proposed a middleware in which the user’s permission can be changed due to the environment and the frequency in which the user frequent it. This way, the given information will vary based on this environment, and the rules of its context.

Zwitter and Gstrein [15] say that data collection and its use must be done concerning the principle of proportionality and individual’s interests. Their work is based on data collected over the individual’s location and genetic data. Thus, the authors exposed user data principles as: sensitivity, privacy and protection, breaches precaution, ethics. The study of Yesmin and Carter [16] was concerned about the patient data through authorized and unauthorized access. The authors developed a framework that audits this access, although the study was limited as real patient information could not validate the tool. Instead, they used real data and could evaluate the amount of unauthorized/unexplained accesses to the patient’s data.

Islam et al. [17] treated data with CIA triad, so that confidentiality is related to the medical information and its protection against unauthorized users. Their study gathered information on various aspects related to the use of IoT devices in medical care. Thus, they say that policies and security measures must be introduced for data protection when sharing data with users, organizations, and applications. Sun et al. [18] combined cryptography with user privacy and their trust relationship with entities, such as family members, physicians, or his device. Thus, these entities are allowed to access the patient’s protected health information. In Plachkinova, Andrés, and Chatterjee [20], the authors studied mHealth apps and the concern about the use of information, terms of use, and privacy policies. The authors mentioned that it is not clear how the data is managed, neither who gets access to it. They developed a taxonomy in which user data is part of the identity threats, access threats, and disclosure threats.

The concern for privacy regarding the device was found in most papers. In Alsubaei, Shiva, and Abuhussein [21], the IoT device is part of the proposed security taxonomy. As their work concerns about mHealth devices, it is part of the proposed taxonomy’s wearable devices, which embraces numerous sensors. The authors describe potential attacks for these devices, as side-channel, tag cloning, tampering devices, and sensor tracking. In the work of Asaddok and Ghazali [11], the authors classified mobile devices as part of the application dimension of the taxonomy, present in the topic ’patient care and monitoring’, as they are used for observation of the patient.

The work of Silva et al. [13] applies privacy over mobile devices regarding aspects such as the environment. Thus, privacy used on mobile devices is part of their taxonomy and a base point of their study. Leithardt et al. [14] are guided on device privacy. This topic is the central part of their work. Zwitter and Gstrein [15] mention mobile devices, although their concern focuses on apps and location data, not the device itself. Islam et al. [17] treat devices like mobile, connected to the Internet through IoT providers. Thus, they are vulnerable to security attacks, which may originate within or outside the network. The authors mention that IoT health devices are part of an attack taxonomy, including information, host, and network. Sun et al. [18] define the Private Device (P-device) as an entity involved in the HCPP system, such as smartphones or wearable devices. The patient uses the P-device to manage privileges on access to his health data. In Plachkinova, Andrés, and Chatterjee [20], the device must be secured, as it can leak data about the location or sensor of the patient. As the apps mentioned in their work fail to provide accurate data management information, the device can be a tool for misusing information.

The use of the data acquired from different sensors needs the implementation of several privacy and security rules. In [25] is presented a low-cost system that embeds the measurement of temperature, heart rate, respiration rate and other parameters to define the health state of the person. This system performs the networking with the healthcare professional to prevent several situations. In addition to these sensors’ data, it includes the tracking of the location of the user to present several contagious. This system may be used for a preliminary diagnosis. Mobile devices are capable of acquiring different types of data in several conditions. Spain was one of the fustigated countries with this pandemic’s situation, and the authors of [26] proposed the implementation of online sensing networks to provide social quarantine and reduce the contagious with the virus.

The monitoring of the COVID-19 needs the use of secured technologies, and the IEEE 802.11ah technology was used in [27] to support the prevention of the contamination with COVID-19. It can be implemented in telemonitoring technologies to provide reliable information and prevent the contact. The network should previously know which are the persons that are contaminated with the virus. The tacking of the location and movements may be performed with location, inertial, and proximity sensors that communicates the data to social networks to reduce the social contact with infected individuals. The authors of [28] studied different privacy constraints related to the real-time monitoring with the mobile devices. The monitoring with mobile devices can be considered to be a digital vaccine that help in the reducing number of contagious with massive sharing of the data.

The creation of a taxonomy was proposed by most of the related works. Alsubaei, Shiva, and Abuhussein [21] proposed a taxonomy regarding IoT layer, intruder type, compromise level, impact, attack method, CIA compromise, attack origin, attack level, and attack difficulty. As can be seen, the taxonomy embraces the security and privacy aspects of medical IoT devices. Barker et al. [10] explored three dimensions to develop a taxonomy, based on visibility, granularity, and purpose. These three dimensions focus on privacy aspects, where visibility deals with who is permitted to access the data. Granularity is focused on the characteristics of that data to direct it to the appropriate use and a dimension that deals with the data’s purpose. In the work of Asaddok and Ghazali [11], the authors developed a taxonomy containing usability, security, and privacy aspects to mHealth applications. Each item of the taxonomy is derived in three or more sub-items. Silva et al. [13] developed a taxonomy or notifications on mobile devices, including communication protocols, message transmission technologies, privacy, and criteria. Plachkinova et al. [20] proposed a taxonomy for mHealth apps regarding security and privacy. The items involve app dimension, security dimension, and privacy dimension.

Table 1 presents a comparison with the related works concerning the application of the privacy aspects described above with the additional taxonomy application.

Even though cryptography is one of the main concerns when dealing with data privacy, descriptions of how to apply it were found explicitly only in the works of Islam et al. [17], Sun et al. [18] and Riza and Gunawan [27]. As Horst Feistel [29] said almost 50 years ago: “personal data needs protection, which can be achieved when enciphering the material”. Cryptography will prevent the plaintext from being accessible to people who are not authorized to have it, whereas it is an important tool when dealing with personal data. The work of Islam et al. [17] comprises a survey of IoT in health care, including analysis regarding the security and privacy aspects. However, the authors did not expose how cryptography can be applied, instead, mentioned that some parts of the flow can be tampered by attackers to obtain the cryptographic secrets. This way, IoT systems should be designed with protections against stealing of cryptographic keys.

**Figure 1:**
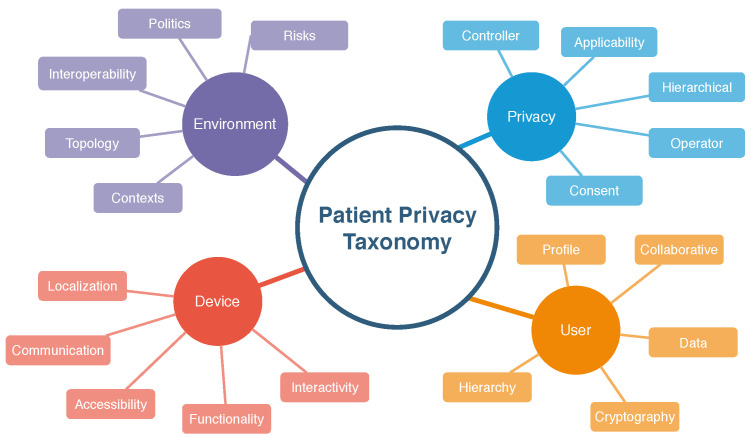
Proposed taxonomy.

The work of Sun et al. [18] is focused on cryptography, as it describes a system based on this aspect. The authors designed protocols for a healthcare system in which the security aspect leverages on cryptographic tools. The HCPP allows the patient to store their medical record even on public servers, where only the patient can retrieve the information. The patient’s medical record is encrypted to ensure privacy, and its content can only be retrieved by the patient and the physician when some treatment is being carried out. If by any means the patient is unable to retrieve the medical record, the system can provide the relevant information to the physician without compromising the patient’s secret key. In our work, cryptography is used to prevent unauthorized access to the patient’s medical records. As it can be seen in our proposed taxonomy in Figure 1, cryptography is part of the User’s items, as it is a critical tool to protect the patient data. The patients’ medical records should be stored and transmitted in encrypted ways, in a way that only the personnel who has the authorization and, therefore, the secret keys, can decrypt the data. Therefore, patients’ medical records are encrypted and can only be accessed by the authorized staff.

In comparison to the selected works, ours stands out because it includes the indication of encryption, profile privacy, concerns on device, and the definition of the taxonomy meant to define the theme and scenario of the application more clearly. Our taxonomic definition aims to embrace the necessary aspects to be covered to enhance security measures throughout the patient’s sensitive data. We developed a mobile application to validate the data flow of information from the moment patients are being admitted in the hospital until they are discharged.

The use of a mobile application that implements data privacy parameters related to the data of patients infected with COVID-19 is another contribution of this study. The data of patients may be its location, temperature, history of navigation, among others. Therefore, we consider that contagion can be identified in the first moments spent in the emergency room using basic information on the health status and the monitoring of the feverish state with the use of IoT devices. The degree of privacy applied in each user’s registration process should enable identifying infected patients without the exposure of sensitive data.

To this end, we have developed a taxonomy that highlights how important it is for confidential information to be handled with care. We have included examples of privacy applications in the use of IoT devices to receive, screen, and providing patient care with a focus on the COVID-19 pandemic.

## 3. Taxonomy

We have developed a taxonomic definition for a better classification of the items related to the privacy parameters. A taxonomy is necessary to identify the critical aspects where security measures and policies need to be applied. Based on the goals of this paper and the comparisons made with the related works, we selected the principal parameters to manage privacy, which are divided into other levels to better embrace the desired security aspects.

As presented in works [13,30,31], a taxonomy allows the systematic organization of relevant data in the form of a hierarchy. The keywords and concepts used to define a taxonomy establish parameters throughout the information production cycle, in which distributed professionals can participate in the knowledge creation process in an organized way. This definition covers four parameters for managing privacy standards in hospital settings within the previously defined context. The selected parameters with five attributes were considered necessary for this scenario. Figure 1 shows the taxonomic definitions proposed in this paper.

### 3.1. User Parameter

The user parameter designates the person who provides, controls, or operates the sensitive data used in privacy handling. This parameter refers not only to the patient but also to the participants in the data’s provision or control. For this parameter, we set the following attributes: profile, collaborative, hierarchy, cryptography, data. The profile attribute covers several items that will be part of the process. According to Fengou et al. [32], six entities participate in interactions taking place in the hospital environment:

- the patient himself/herself;
- the clinical network that will care for the patient, including doctors, family members, volunteers, health insurance provider, among other things;
- the hospital;
- smart home as an environment with ubiquitous equipment’s capable of providing security and quality of life;
- the environment in which the patient works, the vehicle with which the patient is transferred to the clinical center.

Based on the entities listed, it can be observed that the user profile is one that must be substantiated, along with the profiles of other entities. The patient’s cooperativeness in providing their registration data is fundamental for a better experience in the given setting. According to Leithardt [33], the user must provide access to their information and services, thus favoring both their expertise in using the service and the system’s improvement whole. The hierarchy enables proper separation of the levels and permissions of each user type. Viswanatham and Senthilkumar [34] proposed the so-called hierarchy-based user privacy, where the information is encrypted and decrypted based on access levels and releases.

The General Data Protection Regulation (GDPR) deals with the need to protect confidential data and the inevitable risk of data theft. Encryption reinforces that all sensitive information must be covered by an acceptable security level, either at its source or at its destination. Ibraimi et al. [35] said that patient confidentiality is one of the significant obstacles in obtaining medical data, as some information is not shared for fear of it being saved in databases that do not comply with security regulations. The protection of sensitive patient information is an essential task. The Department of Health and Human Services, 2002 (HIPAA) Privacy Standard [36] deals with the security of sensitive patient information in the medical field. It is a US federal law created in 1996 to impose standards for protecting such information and preventing it from being shared without the patient’s consent. Cooper et al. [37] deal with privacy and security in data mining in the medical field and cites HIPAA in information privacy matters. In 2002, they suggested that protective measures be imposed by health plans, clinical centers, and other entities involved.

### 3.2. Environment Parameter

The environment parameter represents the smart physical location where user data will flow between different systems and devices. For this parameter, we define the following attributes: topology, interoperability, policies, risks, hierarchy. Topology refers to the architecture of a hospital environment. Costa [38] comments that hospitals used to be built with an emphasis on the utility of the building and the technique used. The health field’s processes and dynamics are often determined by how the wards, sectors, and departments that house distinct functions are arranged. In many of the methods that occur during the patient’s journey through the emergency room, one or more systems are used.

Interoperability between systems is strongly present in the medical field presently. According to Lopes [39], strategies used to be designed and developed from an internal perspective of organizations, with no motivation for integration with other systems. In all the smart environments that people transit, data is shared between information systems and IoT devices. The data are a vital part of the operation of a health institution. Several policies need to be established to apply access security to these environments and define what data will be exchanged between systems and devices. According to Yildirim et al. [40], information security management is an activity that aims to implement a set of policies that help to define an acceptable level of security in these environments, minimizing the potential risks inherent in the exploitation of this information.

Risk management in hospital settings is a crucial activity for the proper functioning of the operation. According to Florence et al. [41], the risk is an estimated value that considers the probability of occurrence of damage and the severity of said damage. Therefore, procedures are meant to minimize those factors that need to be mapped, controlled, and defined. The dimension in the patient’s care is large and complex. It occurs at various times and in multiple environments in the course of service, along with several interactions between the patient, other participants, and technologies. Soares et al. [9] emphasize that due to its characteristics and complexity, the hospital environment favors establishing power and asymmetrical relationships between the nursing team and patients. The asymmetry results from the patients’ fragility and vulnerability in the face of health-diseases processes.

### 3.3. Privacy Parameter

The privacy parameter designates how each piece of information will be handled according to its characteristics. For this parameter, we define the following attributes: communication, applicability, controller, consent, operator. The transmission is linked to the type of user profile and will usually involve unsafe transmitting the information. According to Machado [42], anonymization or encryption in particular pass through the means of communication, i.e., the very existence of communication drives the need to apply security measures to data. It is a basic human right to have one’s sensitive data handled with care. Thus, its applicability is significant. The General Data Protection Law (LGPD) [43], as the Brazilian Data Protection Law, aims to apply standards and laws regulating and protecting individuals’ data. Without this application of standards and regulations, sensitive information could easily be used by those who should not have access to it in the first place.

A categorization determines who has the authority to decide the type of treatment that personal data will be submitted. As mentioned in the LGPD [43], the controller must obtain the consent of the individual owner or holder of the concerned data. The user may, in turn, deny or grant access to their information by a third party. The user must give their consent, a manifestation by which they agree that their information be used in a specific way for a particular purpose. As mentioned in the LGPD [43], if the controller wishes to use this data at another time, consent will be requested once more. The operator shall be responsible for carrying out the data processing determined by the controller. As mentioned in the LGPD [43], the operator is jointly and severally liable for the damages caused by data handling if the strategy does not comply with legal provisions or is not in line with the controller’s instructions. The user provides their consent, and the operator is responsible for processing the information made available when for personal use or transfer to third parties.

### 3.4. Device

**Figure 2:**
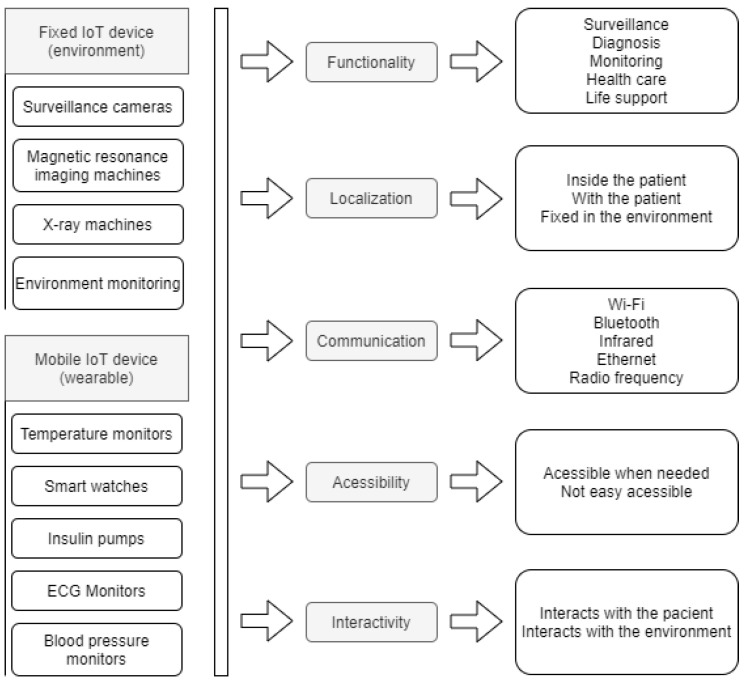
Devices with its parameters.

The device represents either the IoT equipment present in the smart environment that will interact with the patient’s data or the wearable IoT device that will be set to monitor the patient’s temperature. There may be devices that are fixed in the environment, such as surveillance cameras or devices that can be used to monitor the patient, which can be fixed or mobile. For this parameter, we define the following attributes: function, location, communication, accessibility, interactivity. The device must meet the needs of the process to which it will be directed. According to Lupiana and O’Driscolle Mtenzi [44], one of the relevant requirements for devices is their storage and processing capacity. The location attribute refers to the location where the device is installed. For Leithardt [14], the attribute that controls location must be linked to a database where all user data must be included. This database will be accessible only for updating and validating some data. The other information should be processed from the point where the user has accessed the system to provide greater security and reliability. Figure 2 shows both fixed and wearable IoT devices and how the parameters are applied. For both fixed and wearable IoT devices, all five parameters are used. The last column on the Figure 2 shows some of the possible options for each attribute.

The way the device communicates with the user is addressed through the communication attribute and fits in heterogeneity, a feature that ensures information is handled evenly. According to a study presented by Pradilla, Esteve, and Palau [45], the devices are responsible for taking data acquisition through sensors, supporting data treatment with processing units, and acting in conjunction with IoT. Therefore, it is necessary to use heterogeneity in the communication protocols handled by the device and the number of services and types available. This attribute is associated with the protocols of the device, providing security in data transfer. The possibility to access the device whenever necessary is crucial, and interactivity between the device and the client must be ensured. With this in mind, we have developed a model based on the characteristics and functionalities defined in the described taxonomy.

## 4. Project Modeling

The model consists of use case diagrams, sequence diagrams, and context diagrams. All these notations are based on UML. The described model refers to the process from the patient’s arrival at the hospital until his discharge.

### 4.1. Use Cases Diagrams

**Figure 3:**
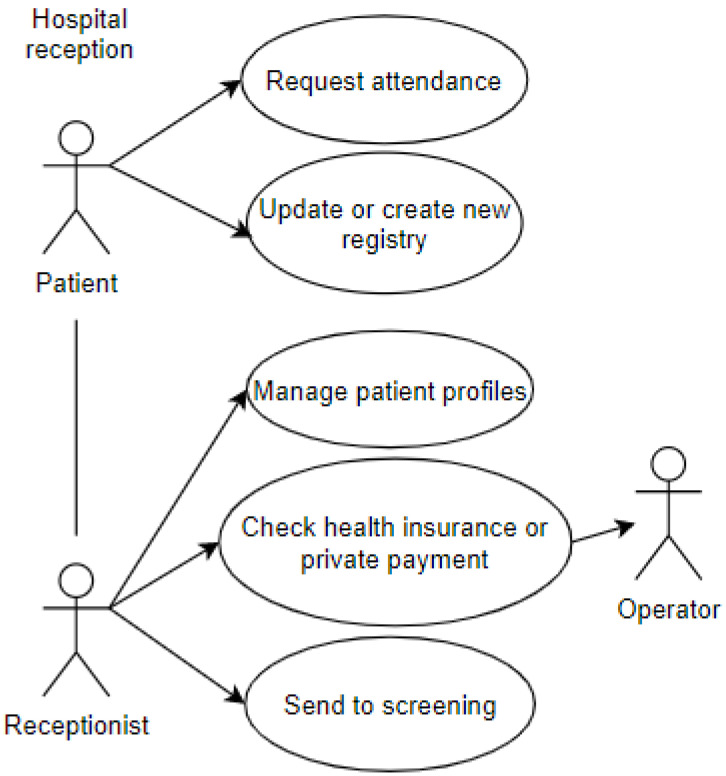
Reception at the Emergency Room.

The first use case represents the entry of a patient into the emergency room. The patient interacts with the receptionist and performs some procedures. This use case includes some of the attributes of the proposed taxonomic definition: privacy, represented by the data which the patient grants access to and is registered in the systems; user, represented by the patient and the receptionist; environment, represented by the emergency room, shown in Figure 3.

**Figure 4:**
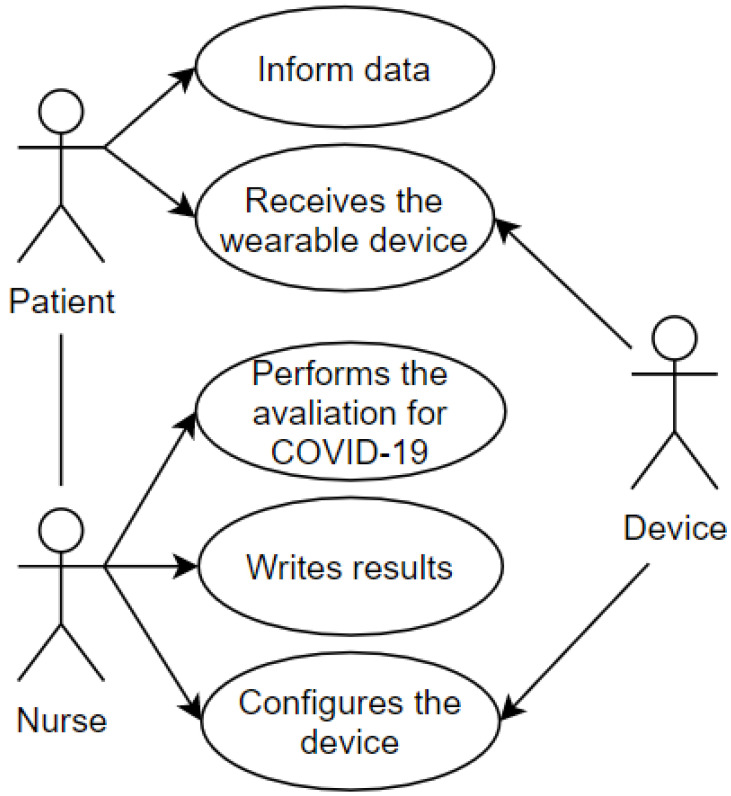
Screening Room.

After first care and registration, the transfer of the patient to the screening area is demonstrated in the use case pictured in Figure 4. The screening process aims to establish the urgency of the case and the risk classification. This use case includes some of the attributes present in the proposed taxonomic definition: privacy, represented by the data which the patient grants access to and is registered in the systems and the wearable IoT device; user, represented by the patient and the nurse; environment, represented by the screening room; device, represented by the wearable IoT device that will receive an identification to record the data and the classification of this patient.

**Figure 5:**
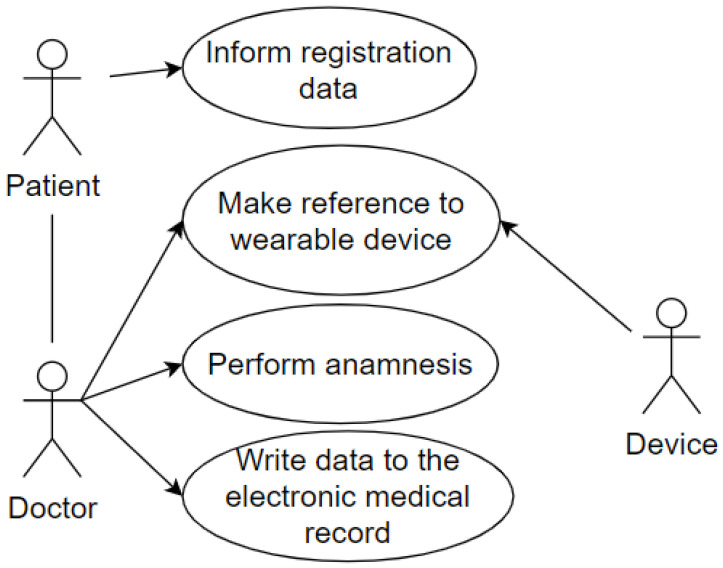
Reception at the Office.

And the last use case represents the patient being attended to by the doctor in the office after going through the screening process. The wearable IoT device identifies the patient so that the data is made available, and the doctor proceeds with the consultation. The doctor performs the anamnesis and records the data in the Electronic Health Record (EHR). This use case uses some of the attributes of our taxonomy as follows: privacy, represented by the data which the patient grants access to and is registered in the systems; user, represented by the patient and the doctor; environment, represented by the office; device, represented by the wearable IoT device used by the patient. This case is illustrated in Figure 5.

Sequence diagrams of each use case were also developed. Sequence Diagram is a UML tool used to represent interactions between objects in a scenario, performed through operations or methods.

### 4.2. Sequence Diagrams

**Figure 6:**
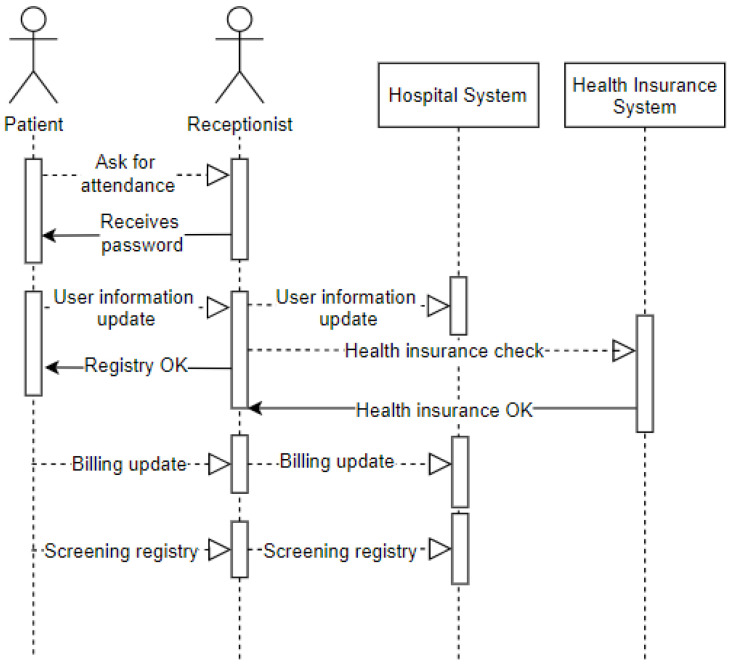
Sequence—Reception at the Emergency Room.

The sequence diagram displayed in Figure 6 represents the entry of a patient into the emergency room. It demonstrates the arrival of the patient (user) to the emergency room (environment), where they request assistance from the receptionist (user). The receptionist provides a password to the patient waiting to be called on. Upon being called on, the patient offers data for registration updates (privacy) recorded by the receptionist in the hospital system. The receptionist checks if the patient has a health plan and then records how this service’s billing issue will be managed. After this procedure, the patient will be referred to as screening.

**Figure 7:**
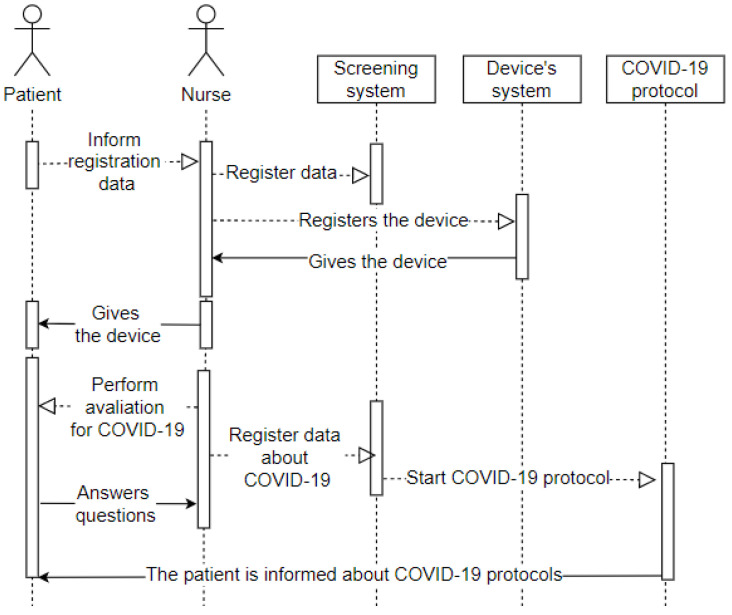
Sequence—Screening Room.

The sequence diagram displayed in Figure 7 represents the patient’s entry into the screening room after completing the first stage in the emergency room. It means the arrival of the patient (user) to the screening room (environment), where they will convey their data as requested by the nurse (user). The nurse records the hospital system’s data and the entry into the system that configures the wearable IoT device that will monitor the patient (device). The receptionist then hands the wearable IoT device over to the patient and starts the assessment. The patient answers the questions (privacy), and the nurse records all the information in the hospital system. All patient data is in the system, and the hospital from their wearable IoT device can track it.

**Figure 8:**
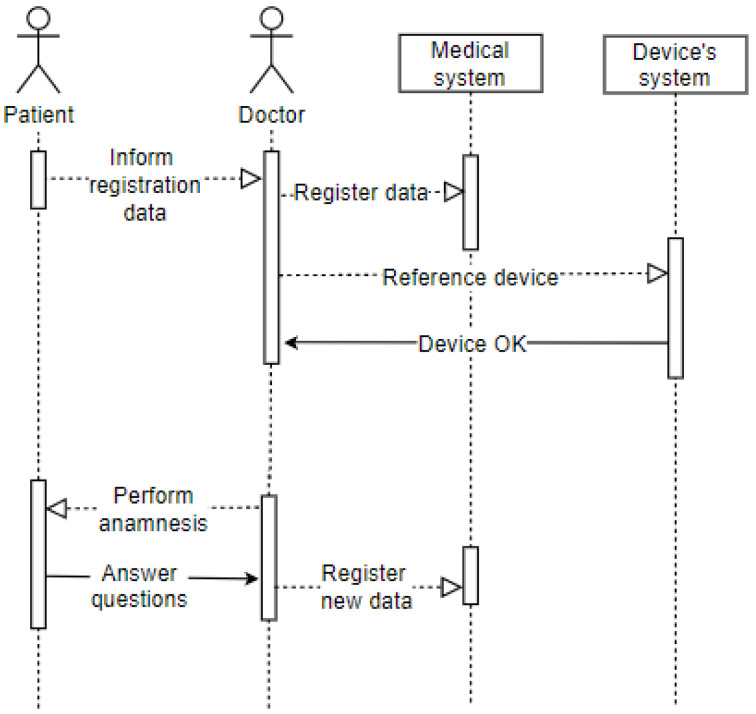
Sequence—Office.

The sequence diagram displayed in Figure 8 shows the patient’s entry into the office after going through the screening process. It represents the arrival of the patient (user) to the office (environment), where they will convey their identification data as requested by the doctor (user). The latter records the electronic record data and refers to the patient’s wearable IoT device in the hospital system. The doctor performs the anamnesis on the patient, who must answer the questions (privacy). The doctor also records this information in the patient’s electronic record. The patient has already been attended to, so they are drugged and released or referred to another hospital ward based on the clinical condition’s evolution.

### 4.3. Context Diagrams

**Figure 9:**
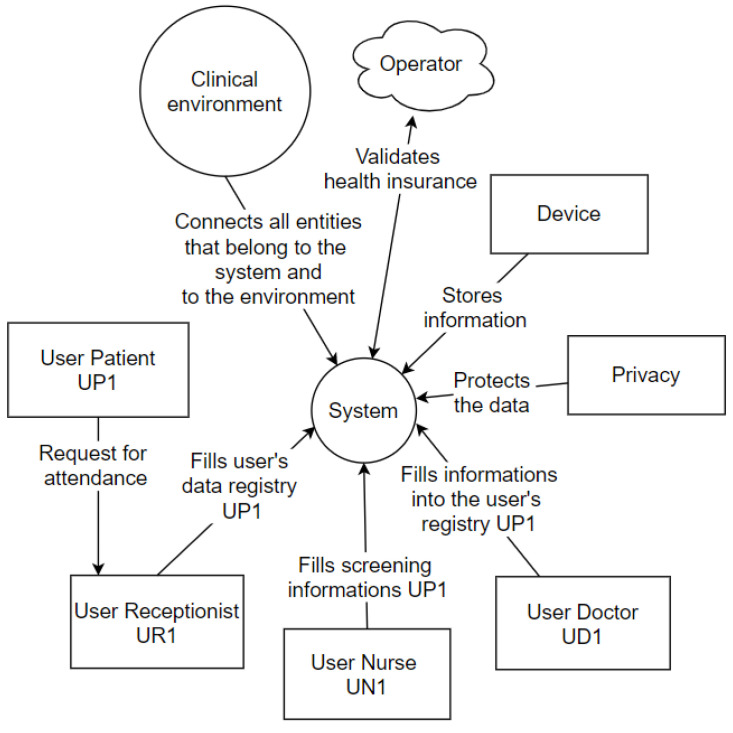
Context Diagram.

The Context Diagram is a UML tool that represents the entire system as a single process. It consists of data streams that show the interfaces between the system and external entities [46]. The diagram illustrates the object of the study, the project, and its relationship to the environment. Figure 9 represents the context diagram of this project.

The patient (user) requests assistance from the receptionist (user), who will fill in the data (privacy) in the hospital system. The hospital system interacts with the operator’s system that is outside the physical environment of the hospital. In the screening process, the nurse (user) conducts the questionnaire with the patient (user), entering the basic health data in the central hospital system, which interacts with the wearable IoT devices system. Finally, the doctor (user) performs the anamnesis, entering the central hospital system’s consultation information. These information registration processes are focused on privacy determinations, and all processes occur in a clinical setting, explicitly represented within the context diagram.

## 5. Prototype

A mobile application was developed as a prototype to illustrate the basic principles, from the admission of the patient to the emergency room and referral to the office or discharge indication. The goal of the developed mobile application is to validate some taxonomy items, for it embraces the environment (interoperability among the system and the wearable IoT device). The application was developed using NodeJS.

**Figure 10:**
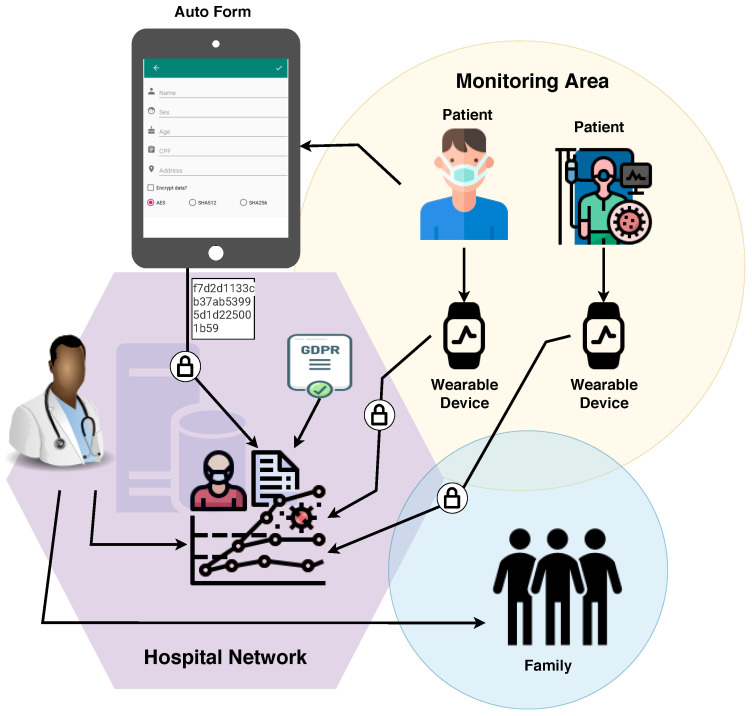
Application Flow.

The application comprises an initial customer registration screen, which simulates the process of filling out the registration form upon admission to the emergency room. The prototype only contains the primary fields: name, gender, age, and address. The ’encrypt data?’ checkbox has been included to select the encryption/hash algorithm. Since it is merely a prototype for demonstrating the flow of information and its security application, the hashes SHA-256 and SHA-512 were made available. In the real application, they would not serve to encrypt data because hashes are not reversible and are considered a one-way function [47]; the prototype also includes the Advanced Encryption Standard (AES) symmetric encryption algorithm. Figure 10 illustrates the first registration screen of the application with the fields mentioned above.

As shown in Figure 10, the application’s flow is as follows: initially, the patient fills out a form with personal data. The data is encrypted and sent to the systems through the hospital network, as necessary. The patient, then, is sent to the screening room to answer more questions and thus help medical personnel assess their situation. He receives a wearable device to monitor his health status. All the information collected about the patient and their health status is included in their digital record. If communication with other systems is required, the information to be sent is encrypted.

The link between this device and the patient’s file allows the information to be collected without a health professional’s intervention. Based on the information provided by the wearable, the system makes a temperature analysis. If the patient remains in a feverish state, they are referred to the doctor’s office. Since fever is one of the symptoms that prevail in detecting COVID-19, its absence can prompt a discharge. However, the lack of fever is not a guarantee that there is no infection with the virus [48], so careful monitoring is needed. In addition to the factors described, comparative tests were performed to validate the application based on the initially defined requirements in the taxonomy.

### Pseudo-Code

The algorithms applied in the development of the prototype application are described below in pseudo-code format. Pseudo-code covers the generation of a service number, temperature monitoring, and referral in case of emergency.

Algorithm 1 deals with the generation of the service number, where the patient’s data will be saved in an encrypted form and forwarded to the monitoring room. In the monitoring room, the service number to be linked to the customer will be generated.
**Algorithm 1:** POST New medical care1 Service Number; **Output**: Attendance number2 save encrypted packet data;3 send to monitoring room

Algorithm 2 deals with the process of monitoring temperature. The wearable IoT device collects the patient’s temperature during the period defined by the medical team and sends it to the server for the monitor process. First, if it is higher than 38.5 °C, the patient is referred to the ICU. If it is equal to or above 37 °C for five minutes, the patient is referred to another ward for medical assistance. Finally, if it is less than 37 °C for ten minutes, the patient can be released.
**Algorithm 2:** Monitoring
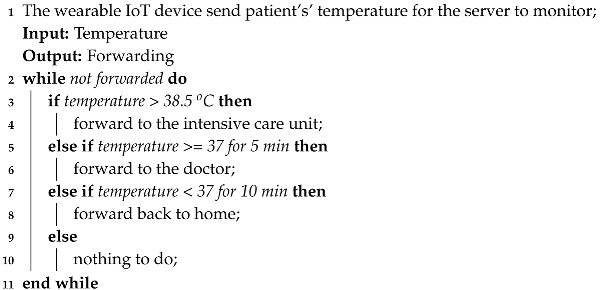


Algorithm 3 deals with the alert generated for the ICU in cases where the patient is classified as an emergency. If there is no emergency, the alert is generated for the doctor, informing that the patient will be referred for care.
**Algorithm 3:** Alert
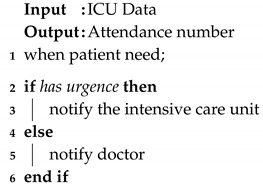


## 6. Tests and Results

**Figure 11:**
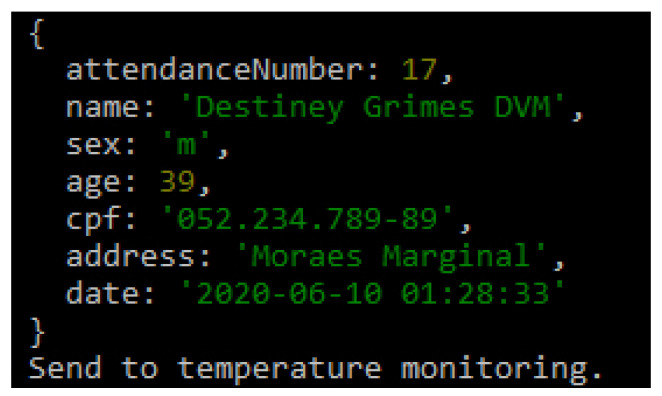
Saved Information.

The flow of controlled information in the application starts after the registration data has been filled in; it is also possible to apply other requirements such as encryption to the patient’s data. Figure 11 illustrates the integration of basic patient information and reports that the patient was sent for temperature control. The temperature was captured and sent to the system, which will classify the feverish state, suggesting different referrals for each scenario. If the patient exhibits a feverish state and has other symptoms that may characterize COVID-19, their care must be provided in a differentiated way.

**Figure 12:**
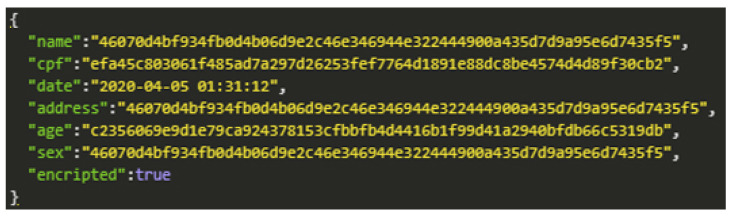
AES Encryption.

When choosing the type of encryption in the data registration process, the data security level increases, and the information should only be made available to those who have permission. For prototype demonstration purposes, we use the AES symmetric key encryption method. The encryption application aims to secure data while transferring it to other devices. Figure 12 shows the encrypted patient registration data.

**Figure 13:**
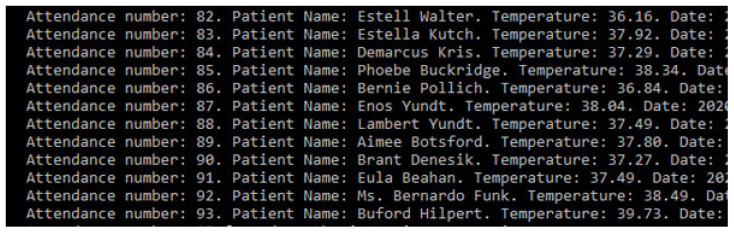
Patient record simulating discharge.

After the patient has been registered, and the information is stored safely, the data is sent to a system that continually gets updates on body temperature. With this prototype’s application, we also tested the hypothesis that an IoT device can monitor the patient for changes in temperature. To test the idea, we implemented a set of random values read by the program to simulate this monitoring process. Every minute, the device will check the temperature of the patients who have entered the system and are waiting at the emergency room’s reception. If their temperature can be characterized as feverish, then they are taken to the office with priority. Figure 13 describes the monitoring of a patient whose temperature remains stable, and hospital discharge is suggested.

**Figure 14:**
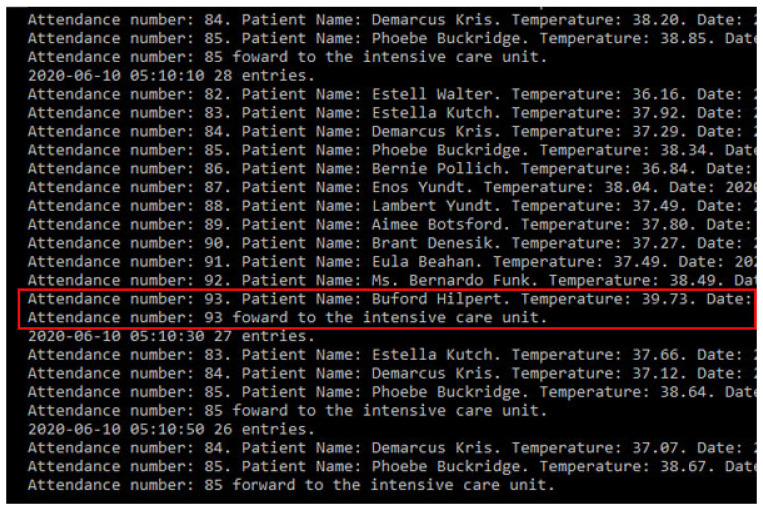
Patient registry simulating medical care admittance.

If the patient’s state remains feverish for five minutes, a message will be sent to the doctor in charge, as shown in Figure 14. If the temperature remains stable for ten minutes, the patient will be released.

After testing and validating the application, it was possible to observe that the information flows through different devices. For the simulation environment, we experimented with only one system that communicates with a wearable device. In real applications, there could be more than one device interacting with more than one method. However, the information’s fluidity would be similar: the patient’s registration at the time of admission to the emergency room, the system being accessed by the screening sector to insert health status data, and the information is received from monitoring devices. At the medical consultation time, the system would receive more details regarding anamnesis, referrals for exams, or hospital discharge.

Given that the feverish state is strongly associated with a COVID-19 diagnosis, the patient should be monitored continuously and receive adequate care as long as the symptoms persist. The high contagion of the virus makes such care essential. The monitoring interval parameters, indicative of medical discharge or a possible disease carrier, are defined according to medical protocols. We emphasize that the interval and discharge suggestion present in this work are meant to simulate features.

## 7. Conclusions

The COVID-19 scenario requires particular solutions for providing the emergency care process and security in the data generated in all environments. In this sense, this work proposed a taxonomy that was designed to support the development of privacy mechanisms for health environments.

The taxonomy is branched into four items containing five attributes each; all the items and their respective attributes are justifiable. For the information flow tests, we developed a prototype and application that addresses the main questions about data privacy despite being simple. The application was developed with registration data inputs and different encryption/hash to be applied according to environmental criteria. The application communicates with a wearable that monitors the patient’s temperature and provides treatment in line with the patient’s feverish state, guiding the referral to the doctor’s office or the possibility of discharge. With the application of taxonomic definitions and the agility of medical professionals in the care of patients with suspected COVID-19, the registration data is kept confidential through encryption and privacy requirements. Temperature monitoring should be continuously done; in the case of feverish states that persist for a period defined by the entity and other symptoms suggestive of the disease, the system suggests the patient’s referral without exposing personal data.

The main contribution of this research consists of the analysis of different privacy parameters with a mobile application that considers the different rules proposed in our taxonomy. There is no concrete analysis previously performed that analyzes the privacy constraints with a mobile application. Mobile technologies are commonly used by people, and it may help in the prevention of COVID-19. In addition, more search should be performed, and the taxonomy developed may be improved to be adapted with the real world.

We believe that the research we have carried out contributes to several other studies currently in progress in several countries, which propose monitoring without consent and put forward definitions of use and data privacy criteria. For future work, we are developing improvements for privacy requirements that can be adapted to different countries, thus expanding variable monitoring features to identify patients with COVID-19 and obtain new tests and results.

## Figures and Tables

**Table 1:** Scope of related works

Work	Cryptography	Private Profile	Devices	Taxonomy
[12] (2007)		•		
[10] (2009)		•		•
[20] (2015)		•	•	•
[17] (2015)	•	•	•	
[18] (2015)	•	•	•	
[11] (2017)		•	•	•
[21] (2017)			•	•
[13] (2019)		•	•	•
[14] (2020)		•		•
[15] (2020)		•	•	
[16] (2020)		•		
[25] (2020)		•	•	
[26] (2020)		•	•	
[27] (2020)		•	•	
[28] (2020)		•	•	
Proposal	•	•	•	•

## Abbreviations

The following abbreviations are used in this manuscript:

AES	Advanced Encryption Standard
CIA	Confidentiality, Integrity and Availability
CPF	Cadastro de Pessoa Física
COVID-19	Coronavirus 2
COVID-19 SARS-CoV-2	Severe Acute Respiratory Syndrome Coronavirus 2
EHR	Electronic Health Record
GDPR	General Data Protection Regulation
HIPAA	Department of Health and Human Services, 202l
HCPP	Healthcare system for Patient Privacy
ICU	Intensive Care Unit
IoT	Internet of Things
LGPD	General Data Protection Law
mHealth	Mobile Health
P-device	Private Device
UML	Unified Modeling Language
